# The Potential Roles of Unique Leaf Structure for the Adaptation of *Rheum tanguticum* Maxim. ex Balf. in Qinghai–Tibetan Plateau

**DOI:** 10.3390/plants11040512

**Published:** 2022-02-14

**Authors:** Yanping Hu, Huixuan Zhang, Qian Qian, Gonghua Lin, Jun Wang, Jing Sun, Yi Li, Jyan-Chyun Jang, Wenjing Li

**Affiliations:** 1 Qinghai Provincial Key Laboratory of Qinghai–Tibet Plateau Biological Resources, Northwest Institute of Plateau Biology, Chinese Academy of Sciences, Xining 810008, China; yphu@nwipb.cas.cn (Y.H.); zhanghuixuan19@mails.ucas.ac.cn (H.Z.); q884199@163.com (Q.Q.); wangjun215@mails.ucas.ac.cn (J.W.); qidian1518@sohu.com (J.S.); 2 University of Chinese Academy of Sciences, Beijing 100049, China; 3 School of Life Sciences, Jinggangshan University, Ji’an 343009, China; lingonghua@jgsu.edu.cn; 4 Department of Horticulture and Crop Science, The Ohio State University, Columbus, OH 43210, USA; 5 Scientific Research and Popularization Base of Qinghai–Tibet Plateau Biology, Qinghai Provincial Key Laboratory of Animal Ecological Genomics, Xining 810008, China; 6 Key Laboratory of Adaptation and Evolution of Plateau Biota, Northwest Institute of Plateau Biology, Chinese Academy of Sciences, Xining 810008, China

**Keywords:** *Rheum tanguticum*, Qinghai–Tibetan Plateau, phenotypic plasticity, heteroblasty, 3D leaf shape, leaf-surface temperature, leaf thermoregulation

## Abstract

Leaves are essential plant organs with numerous variations in shape and size. The leaf size is generally smaller in plants that thrive in areas of higher elevation and lower annual mean temperature. The Qinghai–Tibetan Plateau is situated at an altitude of >4000 m with relatively low annual average temperatures. Most plant species found on the Qinghai–Tibetan Plateau have small leaves, with *Rheum tanguticum* Maxim. ex Balf. being an exception. Here, we show that the large leaves of *R. tanguticum* with a unique three-dimensional (3D) shape are potentially an ideal solution for thermoregulation with little energy consumption. With the increase in age, the shape of *R. tanguticum* leaves changed from a small oval plane to a large palmatipartite 3D shape. Therefore, *R. tanguticum* is a highly heteroblastic species. The leaf shape change during the transition from the juvenile to the adult phase of the development in *R. tanguticum* is a striking example of the manifestation of plant phenotypic plasticity. The temperature variation in different parts of the leaf was a distinct character of leaves of over-5-year-old plants. The temperature of single-plane leaves under strong solar radiation could accumulate heat rapidly and resulted in temperatures much higher than the ambient temperature. However, leaves of over-5-year-old plants could lower leaf temperature by avoiding direct exposure to solar radiation and promoting local airflow to prevent serious tissue damage by sunburn. Furthermore, the net photosynthesis rate was correlated with the heterogeneity of the leaf surface temperature. Our results demonstrate that the robust 3D shape of the leaf is a strategy that *R. tanguticum* has developed evolutionarily to adapt to the strong solar radiation and low temperature on the Qinghai–Tibetan Plateau.

## 1. Introduction

Leaves are essential organs for photosynthesis and transpiration of all higher plants. The size and shape of the leaf affect the growth, reproduction, survival and adaptive strategies of plants [1]. In order to adapt to different environments, leaves have high plasticity. Both leaf shape and size show a great variation across biomes [2,3,4,5,6]. For example, the leaves of cacti convert to spines for adaptation to the arid dessert [7]. The leaf area is the most common measure of leaf size [8,9,10]. Leaf sizes vary by over 100,000-fold among plant species worldwide [11]. Across the plant kingdom, leaves vary from less than 1 mm^2^ to greater than 1 m^2^ in area [12]. In general, leaf size has a tendency to decrease with higher elevation, lower annual mean temperature and precipitation, and lower soil fertility [13]. Leaf size is a determinant for leaf thermoregulation because it influences the leaf energy balance between solar radiation warming and transpirational cooling via leaf boundary layers [14].

In addition to leaf size, leaf shape is also a highly plastic trait in plants. Phenotypic plasticity is referred to as the expression of different phenotypes in one species depending on growing conditions [15]; it is crucial for the adaptation and survival of plants [16]. The phenotypic plasticity of the leaves often reflects the balance between the need to maximize the capture of light quanta during photosynthesis and minimizing the damage caused by environmental stresses [4]. However, many phenotypic traits vary as a function of growth and development; furthermore, the growth and development rates themselves are highly plastic [17].

Given the plasticity, leaves can be characterized in different ways, but primarily according to their general shapes (round, ovate, or lanceolate) and margins (smooth, lobed, or serrated) [18]. Leaf shape is highly variable between plant species and individuals. Depending on the overall structure, leaf blades are classified as simple and compound leaves. Interestingly, even leaves from a single genetic individual might have different morphologies. For example, the juvenile leaves of the *Eucalyptus* L’Hér. plants are sessile and ovate, while the adult leaves elongate into petiolated lanceolate [19]. These conspicuous morphological changes in leaves throughout the lifecycle of plants are known as heteroblasty [20]. Indeed, heteroblasty is a phenomenon that results from the temporal development of shoot apical meristems, creating successive changes in the traits of the lateral organs produced at each node, including leaf shape [21,22,23,24]. Many studies have reported on the heteroblasty in various plant genera, including *Hedera* L., *Acacia* Mill., *Eucalyptus* and *Passiflora* L. [20,23,25]. However, there is no report thus far on the heteroblasty of *Rheum* L. plants.

*Rheum tanguticum* Maxim. ex Balf. (Polygonaceae), belonging to the genus *Rheum*, is a perennial herb endangered and endemic to China [26]. The dried roots and rhizomes of *R. tanguticum* are used as purgative and anti-inflammatory agents in traditional Chinese medicine. It is mainly distributed in Qinghai and Gansu Provinces and the eastern Tibetan Autonomous Region at altitudes ranging from 2300 m to 4200 m and can be found on the margins of forests, in valleys or in shrub meadows. It usually takes six years to start flowering. The panicle is large with many small flowers. The fruit is a triangular shape with three swings. The seeds are black and ovoid [27]. Previous studies on *R. tanguticum* mainly focus on chemical components, pharmacological properties and genetic diversity [28,29,30,31]. However, little information is available on the significance of leaf morphology in growth and survival of *R. tanguticum* [32].

**Figure 1:**
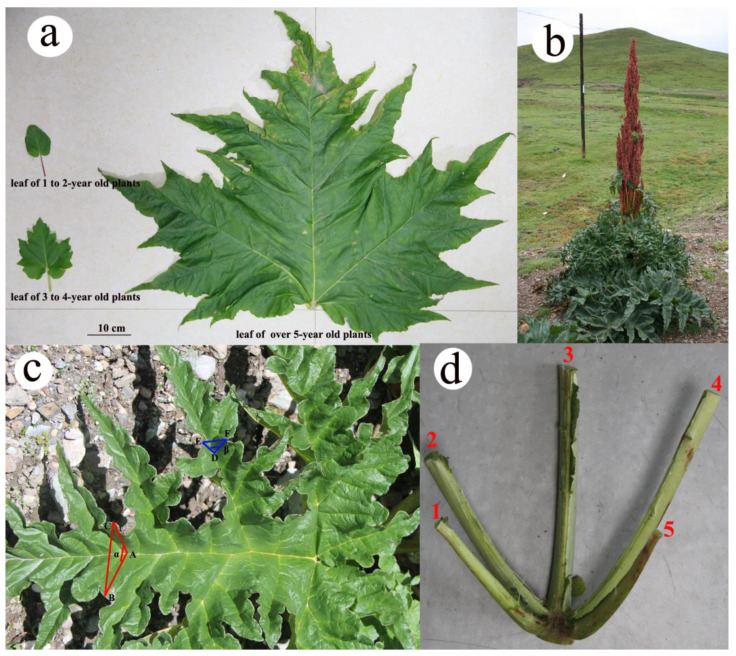
Leaf morphology of *R. tanguticum*: (**a**) Leaves of *R. tanguticum* transitioning from juvenile to adult phase; (**b**) *R. tanguticum* on the Qinghai–Tibetan Plateau; (**c**) Determination of the intersection angle between the blades around the middle of five first-order veins (α) and the intersection angle between blades around the second-order vein (β); (**d**) Five first-order veins in the leaf of an over-5-year-old *R. tanguticum* plant.

The Qinghai–Tibetan Plateau (hereafter QTP) is the highest and largest plateau in the world. Its distinct features include high altitude, low atmospheric pressure, low air temperature, short growing season, high irradiance, strong winds and diurnal temperature fluctuations [33,34]. Maximum viable leaf sizes are especially small in cold and high elevation regions, such as the QTP [11]. Plants with large leaves might be more prone to damage by the high temperatures caused by high solar irradiance [35]. Indeed, the leaves of most plants on the QTP are small, such as species of *Androsace* L. and *Arenaria* L. [36,37], with *R. tanguticum* being an exception. The average plant height of *R. tanguticum* (over-5-year-old plants) is approximately 1.5–2 m tall (Figure 1b). The basal leaves are orbicular or broadly ovate and large. Thus, the obvious question is how could *R. tanguticum* with large leaves adapt to strong solar radiation and low temperature on the QTP? To investigate juvenile-to-adult phase changes in leaf shape, we measured the leaf size, leaf area and intersection angles between the blades. Leaf temperature and physiological parameters of different parts of large leaves were also measured to determine the variation in leaf temperature and physiological parameters within large leaves of over-5-year-old plants. The main objectives of this study are to (1) characterize the heteroblastic changes from the juvenile to the adult phase and (2) determine the roles of leaf morphology and physiology of *R. tanguticum* in adaptation to strong solar radiation and low temperature on the QTP. We hypothesized that the three-dimensional (hereafter 3D) shape of leaves plays an important role in plant thermoregulation in adapting to the unique environment of the QTP.

## 2. Results

### 2.1. Variation in Leaf Number, Size and Shape between Young and Old Plants

The *R. tanguticum* leaves displayed highly significant ontogenetic variations in size and shape (Figure 1a and Table 1; Table S1). Comparing the over-5-year-old to 1–2-year-old plants, large variations were found in the average leaf length and width (>10 times), leaf area (172 times) and leaf dry mass (~400 times) (Table 1). In addition, the leaf number per individual of three age groups changed greatly. There were 1–3 (1.87 ± 0.11) leaves per individual of 1–2-year-old plants, while 4–8 (5.94 ± 0.19) leaves for over-5-year-old plants. The plasticity index of *R. tanguticum* leaf morphology was also calculated (Table 2). Compared to 1–2-year-old and 3–4-year-old plants, the plasticity index of over-5-year-old plants was lower. The plasticity index of the leaf area was the highest in 1–2-year-old plants (0.97) and the plasticity index of leaf dry mass was the highest in both 3–4-year-old (0.83) and over-5-year-old plants (0.79).

**Figure 2:**
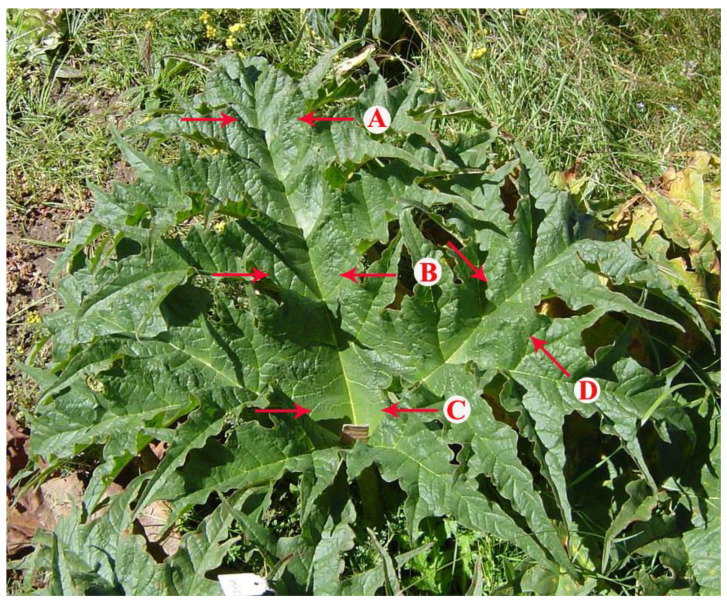
Different temperatures were detected in the leaf blade on the opposite sides of the first-order veins: (**A**) tip of the middle of five first-order veins; (**B**) middle of the third of five first-order veins; (**C**) base of the middle of five first-order veins; (**D**) middle of the fourth of five first-order veins.

In the juvenile-to-adult growth transition, the leaves of *R. tanguticum* changed morphology from small and ovate to large and palmatipartite (Figure 1a and Table 1). There was only a single leaf plane for 1–2-year-old plants. In contrast, each leaf of over-5-year-old plants had more than ten planes, because different first-order veins had different angles relative to the main plane of the middle (#3 in Figure 1d) of five first-order veins. As a result, the leaves of over-5-year-old plants had a complex 3D shape (Figure 1c and Figure 2). Consistent with the observation, the angle between the second of the five first-order veins and the plane of the middle of five first-order veins was 30.60 ± 2.44°, whereas the angle between the first of five first-order veins and the plane of the middle of five first-order veins was 64.20 ± 4.43° (Figure 1d and Table S2).

To further analyze the leaf’s 3D shape in relation to solar radiation exposure, leaf blade intersection angles were calculated by the law of cosines. In over-5-year-old plants, the ratio of BC to AB + AC was 0.50 ± 0.01, which meant that only 50% of the sunlight could be absorbed by the *R. tanguticum* leaves compared to the leaves with the same leaf area completely parallel to the ground (Table S3). Similarly, the ratio of EF to DE + DF (blades around second-order vein) was 0.50 ± 0.01 (Table S4). In contrast, the ratio of BC to AB + AC was 0.68 ± 0.01 in 3–4-year-old plants (Table S5). The leaves of 1–2-year-old plants were entirely on a single plane, so the ratio was approximately 1. In other words, the leaf blade intersection angles decreased rapidly to form a 3D shape during *R. tanguticum* leaf development. According to the measurements, it progressed from 180° (1–2-year-old plants) to 85.44 ± 1.38° (3–4-year-old plants) to 60.51 ± 1.22° (over-5-year-old plants, middle of five first-order veins—α) and 59.65 ± 0.93° (over-5-year-old plants, second-order vein—β) (Table 1).

### 2.2. Leaf Temperature Variation in Over-5-Year-Old Plants

Over-5-year-old *R. tanguticum* large leaves showed a large temperature variation within the same leaf under solar radiation. There were statistically significant temperature differences between the two longitudinal halves of the leaves at the tip regions but not in the middle or basal areas (Figure 2; Figure S1 and Table S6). The greatest temperature differences at the tip, middle and base areas were 8.3, 8.1 and 3.7 °C, respectively. The average temperature differences between the left and right at the tip, middle and base areas were 3.1 ± 0.6, 2.7 ± 0.6 and 1.2 ± 0.3 °C, respectively. There was a significant difference among them (*F* = 4.0, *d.f.* = 2.44, *p* = 0.03). Based on further LSD tests, there were significant differences in temperature variations (left vs. right) between the base and tip (*p* = 0.01) and between the base and middle (*p* = 0.04), but no significant differences between the tip and middle area (*p* = 0.56).

When the ambient temperature was 14.6 °C, the average temperature of the sun-exposed parts of over-5-year-old leaves was approximately 24 °C, while the shaded part was only 17.1 °C (Figure S2), as evidenced by a highly significant difference between them (Table S6). The temperature difference between the sun-exposed and shaded parts of leaves was 7 °C on average, of which 70% (21/30) was more than 5 °C and 17% (5/30) was more than 10 °C. In total, 10% (3/30) of sun-exposed parts of the large leaves could reach above 30 °C. The temperature difference between the sun-exposed parts of the leaves and ambient temperature was 9.4 °C on average, of which 43% (13/30) was greater than 10 °C. Compared to the sun-exposed parts, the leaf temperature of only five individual leaves in the shaded parts were 5 °C higher than the ambient temperature. This indicates that the local leaf temperature change was rather fast upon the transition from sun exposure to shade and vice versa. When the ambient temperature was 17.8 °C, the results were similar to those when the ambient temperature was 14.6 °C (Figure S2) and the highest leaf temperature was 35.6 °C.

**Figure 3:**
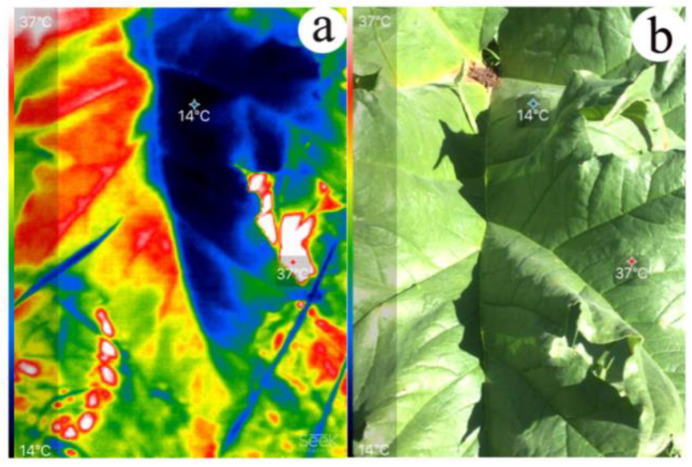
Thermal imaging showing temperature variation within the leaf of over-5-year-old plant of *R. tanguticum*: (**a**) thermal imaging; (**b**) visible imaging.

From the thermal imaging analysis of *R. tanguticum* large leaves, temperature variations between the sun-exposed parts and shaded parts were evident (Figure 3; Table S6). As shown in Figure 3, the highest temperature was 37 °C (sun-exposed parts), while the lowest temperature was 14 °C (shaded parts). By analyzing the thermo-images of 150 over-5-year-old individual leaves, the average highest temperature was 29.3 ± 0.3 °C (with a record of the highest temperature of 38 °C in this study) and the average lowest temperature was 15.9 ± 0.2 °C when the ambient temperature was 16 °C. The differences between the highest temperatures and the lowest temperatures were statistically significant (Table S6). The difference between the highest temperature and the lowest temperature was 13.4 ± 0.3 °C on average, of which 5% (7/150) was less than 10 °C and 5% (7/150) was more than 20 °C.

**Figure 4:**
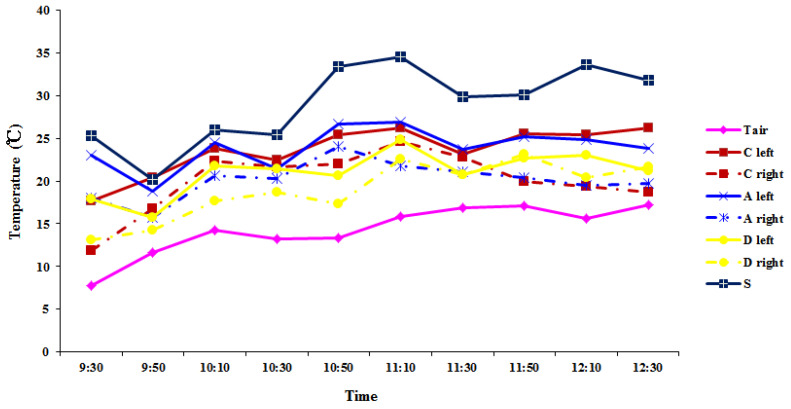
Time-course analysis of temperature change of *R. tanguticum* leaves: Tair, air temperature; S, temperature of small leaves from 1–2-year-old plants; Leaf temperatures from two sides of the first-order vein: leaf tip (A left and A right) and base (C left and C right) of the middle of five first-order veins, and middle of the fourth of five first-order veins (D left and D right) position in over-5-year-old leaf.

To reveal the temporal temperature change within the same leaf, a time-course analysis was conducted to monitor the temperature changes in different leaf positions on a single leaf (Figure 4). As shown earlier, the temperatures of different parts of the leaf were different. However, there was no distinct pattern of temperature change in a given leaf position (base, tip, or middle). Overall, under strong sunlight, the temperature of small orbiculate leaves heated up much faster than the temperature of large leaves of over-5-year-old plants. Hence, the leaf temperatures of small leaves were higher than that of large leaves of over-5-year-old plants. The temperatures in different positions of large leaves were correlated with the ambient temperature (*R* = 0.567, *p* < 0.001; Figure S3). The temperature fluctuation in each position with time appeared to be consistent with the ambient temperature change. The largest increase was 6.1 °C, while the largest decrease was 4.2 °C within 20 min in large leaves (Figure 4). The leaves of over-5-year-old plants were able to buffer the leaf temperature change much more effectively, as evidenced by the higher temperature and greater fluctuation of small ovate leaves of 1–2-year-old plants at each time point. Of note, the time-course experiments were performed on sunny days during a rainy season, when the solar radiation was weaker than average; hence, the leaf temperature variations were smaller than normal.

**Figure 5:**
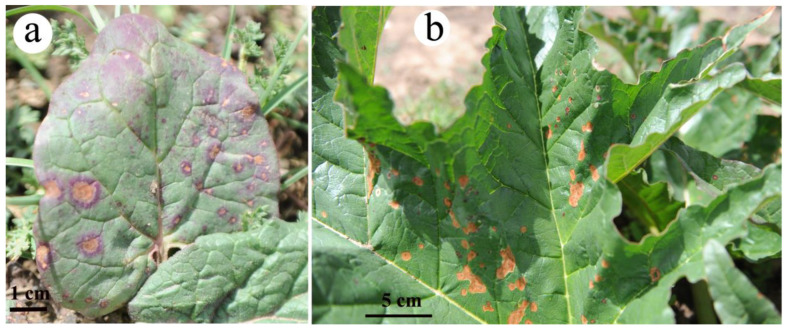
Sun-scorched damages of *R. tanguticum* leaves: (**a**) 1–2-year-old plants; (**b**) over-5-year-old plants.

In addition to leaf temperature variation, we noticed the damages of *R. tanguticum* leaves by solar radiation during our field experiments. The damages to the small ovate leaves could occur randomly without a definite pattern (Figure 5a). Curiously, damages for the large leaves of *R. tanguticum* occurred mainly in the internal parts but not around the edges (Figure 5b).

### 2.3. Physiological Variations within Large Leaves of Over-5-Year-Old Plants

**Figure 6:**
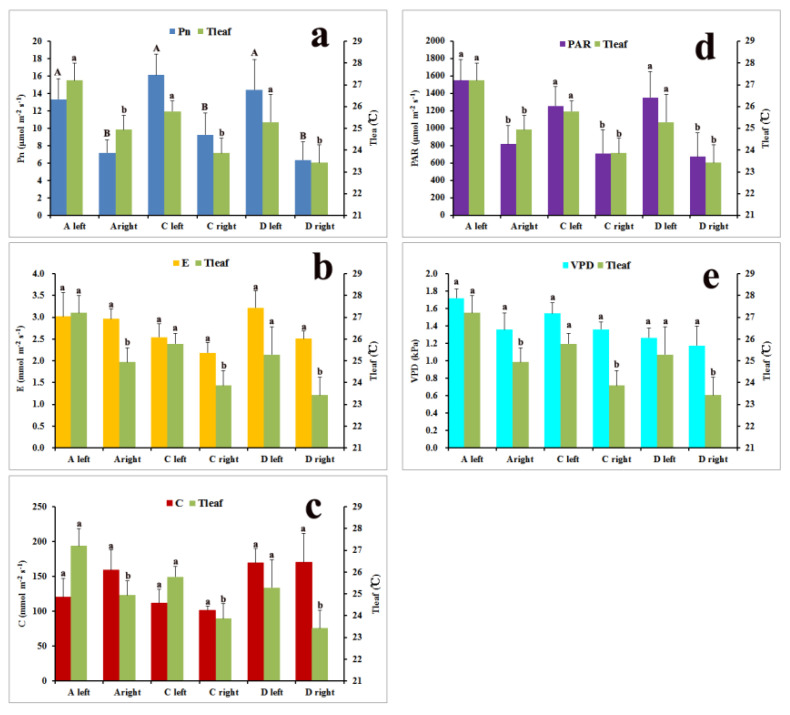
Leaf physiological parameters in different positions of large leaves from over-5-year-old plants (n = 5): (**a**) net photosynthesis rate (Pn); (**b**) transpiration rate (E); (**c**) stomatal conductance rate (C); (**d**) photosynthetically active radiation (PAR); (**e**) vapor pressure deficit (VPD). Tleaf, leaf temperature. Leaf positions on two sides of the middle of five first-order veins are labeled for the tip (A left and A right) and base (C left and C right). Leaf middle positions on the opposite sides of the fourth of five first-order veins are labeled (D left and D right).

The net photosynthesis rate (Pn), transpiration rate (E) and stomatal conductance rate (C) were different at different positions in large leaves (Figure 6; Figure S4). There were no definitive correlations among photosynthetic parameters at different positions on the same leaf. The highest Pn (25.9 μmol m^−2^ s^−1^) was nearly 20 times greater than the lowest one (1.3 μmol m^−2^ s^−1^) on the same leaf. The highest photosynthetically active radiation (PAR) (1995.8 μmol m^−2^ s^−1^) was 40 times more than the lowest one (42.7 μmol m^−2^ s^−1^) on the same leaf. The maximum C (194.7 mmol m^−2^ s^−1^) was six times greater than the minimum C (32.4 mmol m^−2^ s^−1^), whereas the maximum E (4.1 mmol m^−2^ s^−1^) was only two times greater than the minimum E (1.8 mmol m^−2^ s^−1^) on the same leaf (Figure S4). There were significant differences in Pn, PAR and Tleaf between opposite sides of the same position of large leaves, whereas no significant differences were detected for E, C and vapor pressure deficit (VPD) (Table S7).

We then analyzed the correlation between photosynthetic traits and ecological factors (Table S8). The net photosynthesis rate and transpiration rate were positively correlated with leaf temperature (Tleaf) and photosynthetically active radiation (Table S8). However, the stomatal conductance rate was negatively correlated with the vapor pressure deficit. To further analyze the relationship between photosynthetic parameters and environmental factors, multiple linear stepwise regressions were conducted and the optimal regression equation was obtained (Table S9). The regressions were significant for all measurements. According to the results of the regression models, PAR was the main factor that affected the Pn (Y = 2.821 + 0.775PAR; *R* = 0.775). E was positively correlated with Tleaf and negatively correlated with VPD (Y = −4.465 + 1.095Tleaf − 0.951VPD; *R* = 0.877). The effect of the environmental factors on E was Tleaf > VPD. For C, VPD was the main negative factor (Y = −21.609 + 0.541Tleaf − 1.149VPD; *R* = 0.903).

### 2.4. Airflow around the Leaves of Over-5-Year-Old Plants

The average airflow speed in the middle area (approximately 1 cm above the middle of the third of five first-order veins) of the leaves of over-5-year-old plants was 0.35 ± 0.02 m s^−1^, while that in the outside area (10 cm vertical above the middle area) was 0.30 ± 0.02 m s^−1^. There was a significant difference in airflow speed between the middle and outside areas of the leaves of over-5-year-old plants (Table S10).

## 3. Discussion

### 3.1. Heteroblastic Characteristics of R. tanguticum Leaves

In this study, we investigated the structure of *R. tanguticum* leaves in younger versus older plants and determined how the surface temperatures of the leaves varied as the structure of the leaves changed in older plants. In the juvenile-to-adult transition, the leaf undergoes a transformation from a small oval plane to a giant palmatipartite 3D shape (Figure 1). Heteroblasty is a drastic and abrupt change in morphology and physiology that occurs over the lifespan of certain plant species [24]. Therefore, *R. tanguticum* is a highly heteroblastic species, with a notable change in leaf shape during the lifecycle. For comparison, the variation in leaf size across ontogeny in *R. tanguticum* showed a similar pattern to that seen in *Cardiocinum cordatum* (Thunb.) Makino [38]. According to Wright’s prediction [11], the maximum viable leaf size on the QTP should be less than 100 cm^2^. However, the largest leaf area of *R. tanguticum* we measured was 9701 cm^2^, nearly 100 times greater than Wright’s prediction. The leaf size of *R. tanguticum* is much larger than that of the large translucent bracts of the giant alpine plant *Rheum nobile* Hook. f. et Thomson [39,40]. As a result, the leaves of over-5-year-old *R. tanguticum* plants can be considered on record as one of the largest on the QTP. The evolutionary changes in leaf shape and structure have likely provided an adaptive advantage for *R. tanguticum* to thrive on the QTP. The leaf shape change during the transition from the juvenile to the adult phase of the development in *R. tanguticum* is a striking example of the manifestation of plant phenotypic plasticity. In the latter growing years, the plasticity index of *R. tanguticum* leaves stabilized to a lower value.

Previous studies have shown that the vein was essential for the expansion of large leaves in *Rheum rhabarbarum* L. [41] and this was similar in *R. tanguticum* (Figure 1d). In *R. tanguticum* adult leaves, five first-order veins branch from the petiole. Four of five first-order veins (#1, #2, #4 and #5 in Figure 1d) are inclined above the blade, resulting in angles between these four of five first-order veins and the plane of the middle of five first-order veins. The five first-order veins are not in the same plane, which is the basis for the 3D shape of the leaf blade. However, the five first-order veins of *R. rhabarbarum* were in the same plane. The most notable change in leaf shape of *R. tanguticum* was the change from a single leaf plane to a 3D shape. Due to the 3D shape of five first-order veins, the whole leaf appears to be divided into more than ten different orientations.

### 3.2. Leaf Temperature Variation in Leaves of Over-5-Year-Old Plants

The 3D shape of *R. tanguticum* leaves appeared to be a major cause of leaf temperature variation. Because of the high altitude and strong solar radiation on the QTP [33,34], the temperature of large leaves rose quickly under the sun but increased more slowly or even decreased in the shade (Figure 3; Figure S2). The 3D shape leaf’s surface temperatures were predicted to be much more heterogeneous among different leaf locations, which was supported by the thermal imaging analysis of the leaves. Furthermore, there were temporal changes in leaf temperature in the same position of the large leaves during sun exposure (Figure 4). The local leaf temperature change was rather fast upon the transition from sun exposure to shade and vice versa. The highest leaf temperature in our study was 38 °C, which was 22 °C higher than the ambient temperature of 16 °C. Previous studies [42,43,44] have shown that broad leaves can reach up to 20 °C above ambient temperature. According to the thermal imaging in our study, the average highest temperature of *R. tanguticum* leaves was approximately 29.3 °C, which was 13.3 °C above ambient temperature (16 °C). The heterogeneity of sun exposure on large leaves is the basis for leaf-to-air temperature differences [11]. Our findings on the photosynthesis parameters are consistent with this idea. The photosynthetically active radiation and leaf temperature in different parts of the large *R. tanguticum* leaf were different (Figure 6; Figure S4). The 3D shape of large leaves caused a difference in photosynthetically active radiation, which, in turn, led to different temperatures and different net photosynthesis rates on the same blade. This feature is also significant in the areas of the QTP with lower average temperatures. The locally elevated leaf temperature could enhance enzymatic activities, thus improving the photosynthetic efficiency [45]. Local leaf temperatures can rise to temperatures higher than 30 °C; therefore, the photosynthetic efficiency in such parts of the *R. tanguticum* leaves could reach levels seen in tropical plants.

We inferred that, in the leaves of over-5-year-old plants, there were three possible mechanisms to modulate leaf temperature. First, compared to the plane leaf with the same leaf area, there was less solar radiation on a palmatipartite leaf of *R. tanguticum* (approximately 50% decrease in over-5-year-old plants) (Tables S3 and S4). Hence, this would prevent the leaf temperature from rising too fast or too high. There were also angles between the blades and the middle of five first-order veins (Figure 1c). The older the *R. tanguticum* plants were, the larger the leaves; however, the intersection angles between the blades and the middle of five first-order veins also decreased, thus altering the shape of the leaf blade. Compared to the temporary leaf folds of some plants exposed to bright light [46], this represents a permanent partial leaf fold without energy expenditure. Second, temperature variation facilitated the occurrence of local airflow in the leaves of over-5-year-old plants. The temperature variation in different parts of *R. tanguticum* leaves could be a result of local airflow at a small scale around the leaves, which could lower the temperature in the warmer part of the leaves. Some parts of the leaf were directly under sunlight, while other parts were in their own shadow (self-shading; Figure 3) or that of another leaf. Because the light exposure was opposite on the bilateral sides of the veins, it created a larger temperature variation, which favored the formation of air currents circulating around the leaf (Table S10, Video S1). Third, deep lobing may also reduce leaf temperature. Vogel [44] showed that deep lobing not only improved heat transfer but also notably reduced its dependence on orientation. In addition, pinnate compound leaves dissipate heat more effectively than simple leaves [47]. The lobes of *R. tanguticum* are narrow and triangular–lanceolate. The study of morphological variation in *R. tanguticum* large leaves showed that the blade lobes became narrower with higher latitudes and altitudes [32]. With the increase in altitude, solar radiation becomes stronger and the narrowing of the blade lobes could effectively dissipate the heat to reduce leaf temperature.

Under strong sunlight, orbiculate leaves get heated up rapidly. The temperature becomes especially high at the edges of the orbiculate leaves. Consequently, these orbiculate leaves are easily damaged on the edges of the leaves. Previous studies have suggested that damages in the leaf margins of *Alocasia macrorrhiza* L. leaves might occur due to the limited leaf water potential [35]. However, this mechanism appears less suitable to explain the leaf damages observed in *R. tanguticum*. We propose that the 3D shape of the leaf plays an important role in preventing serious damages to *R. tanguticum* leaves by modulating the leaf temperature, which is high enough to enhance photosynthesis but low enough to prevent severe sunburn. More work is needed in the future to determine whether the leaf anatomical structure and specific metabolic processes of *R. tanguticum* also contribute to the resistance to sunburn.

Together, we propose that the 3D shape of leaves plays a key role in plant thermoregulation of *R. tanguticum* for the following reasons: (1) the temperature was highly heterogeneous in leaves of over-5-year-old plants of *R. tanguticum* under solar radiation; (2) the degree of temperature variation was dependent on leaf position; (3) the local leaf temperature change was rather fast upon the transition from sun exposure to shade and vice versa; (4) the rate of temperature change may also be affected by leaf position; and (5) differential leaf temperature change caused local airflow to modulate leaf temperature. Together, the concerted modulation of local leaf temperature makes the large *R. tanguticum* leaf a mosaic ecosystem that is more resilient for growth and survival on the QTP.

### 3.3. Physiological Characteristics within Large Leaves of Over-5-Year-Old Plants

Many plant species in the *Rheum* genus are heliophilous, which can effectively use bright light and have a higher net photosynthesis rate [48,49,50]. In this study, *R. tanguticum* also showed similar photosynthetic characteristics. The highest Pn of *R. tanguticum* was 25.9 μmol m^−2^ s^−1^ and the PAR was 1995 μmol m^−2^ s^−1^ (Figure S4), which is similar to that of *Rheum officinale* Baill. and *R. emodi* Wall [48,51]. Therefore, the compensation of light radiation and temperature in the plateau allowed photosynthesis to be optimized in the *Rheum* plant species.

The significant differences in Pn, PAR and Tleaf between the opposite sides of over-5-year-old leaves indicated that there was heterogeneity in the photosynthetic characteristics of large leaves. This heterogeneity of photosynthesis is a result of the 3D shape of large leaves. The basal leaves of *R. tanguticum* are rosette (Figure 1b) and the direction of petiole determines the light interception efficiencies of different leaves. This explains the differences in the net photosynthesis rates of different parts of the same individual leaves.

Transpiration can decrease the leaf temperature, but the process requires energy and water. In addition to transpiration, plants evolve with various mechanisms to reduce leaf temperature, especially in dry environments [52]. Leaves can also prevent or decrease heat damage using physical structures such as pubescence or even by leaf movement [46,53], but these processes also require energy input. Unlike *Saussurea medusa* Maxim. [54], which has downy bracts to protect tissues from extreme temperature variations, the leaves of *R. tanguticum* are abaxially pubescent. There were no significant differences in the transpiration rate and stomatal conductance rate between the opposite parts of the large leaves. The maximum stomatal conductance rate (C = 194.7 mmol m^−2^ s^−1^) of *R. tanguticum* is much lower than that of *R. nobile* (more than 1000 mmol m^−2^ s^−1^) [39]. The enlarged stomata were essential for the expansion of large leaves in *R. rhabarbarum* [41]. However, the stomata size decreased with the increase in leaf size in *R. tanguticum* (unpublished data). This indicates that *R. tanguticum* modulates the leaf temperature mainly by the 3D shape instead of transpiration. *R. tanguticum* takes advantage of the physical structure of its leaves for thermoregulation, not solely by transpiration. Thus, this mechanism employs the external infrastructure instead of consuming internal energy and water. This may be one of the important reasons why *R. tanguticum* has large leaves but can adapt well on the QTP.

## 4. Materials and Methods

### 4.1. Study Site and Plant Materials

The study was conducted at Dawu, Maqin County, Golog Prefecture, Qinghai Province (34°25′50″ N, 100°17′3″ E; elevation of 3762 m), China. This region is in the eastern Qinghai–Tibetan Plateau, with an annual mean temperature of 0.64 °C and an annual mean precipitation of 561.16 mm. More than 85% of the annual precipitation occurs from May to September. The maximum monthly mean temperature was 9.9 °C in 1971–2000 which usually occurs in July or August [55]. The annual average sunlight is approximately 2576 h and the ≥0 °C annual cumulative temperature is 914.3 °C [56].

*R. tanguticum* plants were identified by Dr. Wenjing Li (Northwest Institute of Plateau Biology, CAS) and voucher specimens (HNWP-337832) were deposited in the Herbarium of the Northwest Institute of Plateau Biology (HNWP), Chinese Academy of Sciences.

### 4.2. Leaf Number, Leaf Size, Leaf Area and Leaf Dry Mass

*R. tanguticum* exhibits dramatic heteroblastic changes in leaf shape (Figure 1a) during the lifecycle of the plant. The leaves of 1–2-year-old *R. tanguticum* are small and ovate. The leaves of 3–4-year-old plants transition to large and palmatilobate leaves. The basal leaves of over 5-year-old plants are orbicular or broadly ovate, large, 30–80 cm long and deeply palmately five-lobed. Around five basal primary veins branch from the terete petiole of basal leaf. Stem leaves are few and smaller than the basal ones (Figure 1).

According to the trait of leaf morphology, three age groups were distinguished (1–2-year-old, 3–4-year-old and over-5-year-old plants). The number of leaves per individual was counted for about 30 individuals in each age group. One healthy leaf per individual and 30 individuals per group were selected. The sizes of the leaves (length and width, Figure 1a) were measured in the three groups (1–2-year-old, 3–4-year-old and over-5-year-old plants) using a flexible ruler (±1 mm). The leaves (without petiole) of 1–2-year-old plants and 3–4-year-old plants were digitally scanned onto a computer and the leaf area was measured using image analysis software ImageJ (National Institutes of Health, Bethesda, MD, USA) [57]. The leaves of over-5-year-old plants were large and of 3D shape and they could not be scanned onto a computer in their entirety. Therefore, they were cut and spliced into rectangles and then measured (Figure S5). The dry mass of the leaves (without petiole, obtained by oven-drying samples for 72 h at 50 °C) was measured using an analytical balance (±0.0001 g; BS224S; Germany).

The phenotypic plasticity index (PI) was calculated for each measured variable as the difference between the maximum and the minimum value divided by the maximum value. PI ranges from 0 (no plasticity) to 1, indicating high plasticity [16].

### 4.3. The Intersection Angle between the Blades

Leaf veins were classified according to branching architecture [58]. There were five first-order veins in over-5-year-old *R. tanguticum* leaves (Figure 1d). The intersection angle between the blades around the middle of five first-order veins (α) and the intersection angle between blades around the second-order vein (β) are shown in Figure 1c. Thirty undamaged leaves from 3–4-year-old plants (for α) and thirty undamaged leaves from over-5-year-old plants (for α and β) were selected. The lengths of AB, AC and BC (for 3–4-year-old plants and over-5-year-old plants) and DE, DF and EF (for over-5-year-old plants) were measured with a ruler (± 1 mm) (Figure 1c). According to the law of cosines, cosα = (AB^2^ + AC^2^ − BC^2^)/2AB × AC; cosβ = (DE^2^ + DF^2^ − EF^2^)/2DE × DF.

### 4.4. Measurement of the Vein Angles Relative to the Plane of the Middle of Five First-Order Veins

Five mature, undamaged leaves were randomly selected for the measurement of vein angles. The blades were removed and only the five first-order veins were kept. The middle of five first-order veins (#3 in Figure 1d) was put on a plane and the angles between the plane and the other two first-order veins (#1 and #2 in Figure 1d) were measured with a protractor [59].

### 4.5. Temperature and Thermal Imaging of Over-5-Year-Old R. tanguticum Leaves

Leaf temperature (±0.1 °C) was measured with a thermometer (HOBO UX100-023 Ext Temp/RH; Onset Computer Corp., Bourne, MA, USA) at six positions (1 cm from the middle of five first-order veins) along the lamina in the direction of the middle of five first-order veins for fifteen leaves of over-5-year-old plants (Figure 2A–C). Meanwhile, air temperature was measured 3 times during the process of leaf temperature measurement (at the beginning, in the middle and at the end). The mean of the three measurements was presented as the air temperature. In addition, the temperatures of the sun-exposed parts and shaded parts of thirty leaves from thirty individual plants were measured.

A Seek Thermal CompactPRO (Seek Thermal Inc., Santa Barbara, CA, USA) with an iPhone 6 s (0.2 m from the leaf) was used to record the thermal images of over-5-year-old *R. tanguticum* leaves. A total of 150 thermal images of each *R. tanguticum* leaf were used to record the highest and lowest temperatures of different parts of the same leaf simultaneously. Measurements were performed on sunny days from 03:00 p.m. to 05:00 p.m. in August 2019.

To capture the time-course temperature changes, the leaf temperatures of different parts (Figure 2A,C,D) of one palmatipartite leaf from over-5-year-old plants were measured every 20 min from 09:30 a.m. to 12:30 p.m. in early August 2020. The temperatures of one ovate leaf from 1–2-year-old plants were measured for comparison.

### 4.6. Measurement of Leaf Photosynthesis

The leaf photosynthesis parameters were measured with a handheld photosynthesis system (CI-340; CID Bio-Science Inc., Camas, WA, USA) from 10:00 a.m. to 03:00 p.m. on clear sunny days in early August 2020. The net photosynthesis rate (Pn), photosynthetically active radiation (PAR), transpiration rate (E), stomatal conductance rate (C), vapor pressure deficit (VPD) and leaf temperature (Tleaf) were measured on different parts (Figure 2A,C,D) of five leaves from five individual over-5-year-old plants. Measurements were performed with a narrow rectangular chamber (65 × 10 mm) under the open system, while the flow rate was set at 0.3 lpm and the time interval was 1 s. In order to use the instrument chamber, we cut and removed the lamina from the outer region while measuring position C. Each measurement was made within 1–2 min and all the measurements of one leaf were completed within 10 min.

### 4.7. Airflow around the Leaves of Over-5-Year-Old Plants

Airflow speed around the leaves of over-5-year-old plants was measured by a thermal anemometer (AR866A; SMART, Hong Kong, China) on a sunny light breeze day from 09:30 a.m. to 12:00 p.m. The maximum airflow speed within 10 s in the middle area (approximately 1 cm above the middle of the third of five first-order veins) and outside area (10 cm vertical above the middle of the third of five first-order veins) of the leaves was made when there were no movements of the leaves to the naked eye. Four leaves were measured every 10 min and 60 paired data in total were recorded.

### 4.8. Statistical Analysis

To determine the variation in leaf morphology among different age groups and the temperature difference (left vs. right) among different parts (tip, middle and base) of large leaves, a one-way analysis of variance (ANOVA) was performed. LSD tests were used to determine pairwise differences between different groups using a significance level of *p* < 0.05. Paired-sample T-tests were conducted to determine the temperature heterogeneity of large leaves and the airflow around the leaves. The relationship between leaf temperature and air temperature during the time-course experiment was analyzed by Pearson correlation. A correlation analysis between photosynthetic parameters and environmental factors was performed using the multiple linear stepwise regression analysis. In addition, paired-sample T-tests were performed to determine the difference among photosynthetic parameters between the left and right sides of the same position of large leaves. The analysis of variance, correlation, regression and T-tests were performed in SPSS (Version 16.0; SPSS Inc., Chicago, IL, USA).

## 5. Conclusions

During the juvenile-to-adult transition of *R. tanguticum*, the leaf shape changed from a small oval plane to a giant palmatipartite 3D shape. This 3D shape of *R. tanguticum* is likely to be the basis for leaf temperature variations. Large *R. tanguticum* leaves were found to maintain temperatures higher than ambient temperatures, which compensates the problem of low ambient temperature on the Qinghai–Tibetan Plateau. On the other hand, the 3D shape of leaves ensures that leaf temperature stays within the physiological range suitable for cellular metabolism and for avoiding sun damage. The large leaf of *R. tanguticum* with its 3D shape is an ideal solution that leads to thermoregulation with little energy consumption. In this study, we propose that the 3D shape of leaves plays a key role in plant thermoregulation. In principle, *R. tanguticum* is highly heteroblastic, with a striking change in leaf morphology during the juvenile-to-adult transition. The 3D leaf shape, resulting in efficient thermoregulation and airflow, is a strategy that *R. tanguticum* has developed to adapt to the unique environment on the Qinghai–Tibetan Plateau. However, our knowledge on the formation and movement of the airflow around the leaves of over-5-year-old plants of *R. tanguticum* is still limited. Further investigation into the details of airflow would help gain insights into the mechanism involved in the thermoregulation of 3D shape of leaves. It is also imperative, for future research on intrinsic gene regulation in the process of leaf development, to elucidate the mechanisms underlying the heteroblasty of large *R. tanguticum* leaves. Finally, more comparative studies are needed on the cues that give rise to the 3D shape of leaves in other plant species in different ecosystems.

## Figures and Tables

**Table 1:** Leaf variation among *R. tanguticum* plants at different ages.

Traits	1–2-Year-Old	3–4-Year-Old	Over-5-Year-Old
Size	Small	Medium	Large
Shape	Ovate	Palmatilobate	Palmatipartite
Leaf number per individual	1.87 ± 0.11c (n = 30)	2.71 ± 0.12b (n = 31)	5.94 ± 0.19a (n = 32)
Length of leaf (cm)	7.63 ± 0.39c (n = 30)	22.02 ± 1.10b (n = 29)	82.79 ± 2.67a (n = 30)
Width of leaf (cm)	6.25 ± 0.36c (n = 30)	19.36 ± 1.23b (n = 29)	86.44 ± 2.81a (n = 30)
Leaf area (cm^2^)	25.50 ± 3.50c (n = 35)	173.27 ± 15.51b (n = 14)	4406.08 ± 671.29a (n = 12)
Leaf dry mass (g)	0.09 ± 0.01c (n = 19)	2.07 ± 0.27b (n = 15)	35.69 ± 6.19a (n = 9)
Intersection angle around middle of five first-order veins (°)	180	85.44 ± 1.38 (n = 30)	60.51 ± 1.22 (n = 30)
Intersection angle around second-order vein (°)	-	-	59.65 ± 0.93 (n = 30)

Lowercase letters represent significant differences between groups.

**Table 2:** Phenotypic plasticity index of *R. tanguticum* plants at different ages.

Plasticity Index	1–2-Year-Old	3–4-Year-Old	Over-5-Year-Old
Length of leaf	0.64	0.63	0.50
Width of leaf	0.69	0.68	0.52
Leaf area	0.97	0.67	0.79
Leaf dry mass	0.91	0.83	0.79

## Data Availability

Not applicable.

## Supplementary Materials

The following supporting information can be downloaded at: https://www.mdpi.com/article/10.3390/plants11040512/s1, Figure S1: Leaf temperatures of 15 individual samples on the opposite sides of the middle of five first-order veins: (A) tip; (B) middle; (C) base, Figure S2: Leaf temperatures of sun-exposed and shaded parts from 25–30 individual samples: (a) ambient temperature at 14.6 °C; (b) ambient temperature at 17.8 °C, Figure S3: Correlation between leaf temperature of the over-5-year-old plant and air temperature during the time-course experiment, Figure S4: Leaf physiological parameters in different positions of large leaves from over-5-year-old plants in detail: (a–e) net photosynthesis rate (Pn); (f–j) transpiration rate (E); (k–o) stomatal conductance rate (C); (p–t) photosynthetically active radiation (PAR); (u–y) vapor pressure deficit (VPD); Tleaf, leaf temperature. Leaf positions on two sides of the middle of five first-order veins are labeled (A left and A right) for the tip and (C left and C right) for the base; leaf middle positions on the opposite sides of the fourth of five first-order veins are labeled (D left and D right), Figure S5: A spliced leaf composite from an over-5-year-old *R. tanguticum* plant, Table S1: Statistical analysis of the leaf characters of *R. tanguticum* at different ages, Table S2: The angles between different veins and plane of the middle of five first-order veins, Table S3: Details of the calculations of intersection angle (α) and ratio of solar radiation exposure in leaves of over-5-year-old *R. tanguticum*, Table S4: Details of the calculations of intersection angle (β) and ratio of solar radiation exposure in leaves of over-5-year-old *R. tanguticum*, Table S5: Details of the calculations of intersection angle (α) and ratio of solar radiation exposure in leaves of 3–4-year-old *R. tanguticum*, Table S6: Summary of statistical analysis of the temperature of opposite parts of leaves, Table S7: Summary of statistical analysis of the photosynthetic parameters of opposite parts of over-5-year-old large leaves, Table S8: Correlation matrix of photosynthetic parameters and environmental factors of *R. tanguticum*, Table S9: Multiple linear regression equations for photosynthetic parameters and environmental factors of *R. tanguticum*, Table S10: Statistical analysis of the speed of airflow around the leaves of over-5-year-old plants, Video S1: Airflow indicated by smoke movement around the large leaves of over-5-year-old plants.
