# Supplementary material for: The Potential Roles of Unique Leaf Structure for the Adaptation of Rheum tanguticum Maxim. ex Balf. in Qinghai–Tibetan Plateau
Source: Plants (Basel). 2022 Feb 14;11(4):512. doi: 10.3390/plants11040512 (PMC8875413; doi:10.3390/plants11040512)
Supplement: Supplementary file 1 [file plants-11-00512-s001.zip › plants-1549688-supplementary without change-0204.pdf]

Supplementary Material

Yanping Hu [1], Huixuan Zhang [1,6], Qian Qian [1,6], Gonghua Lin [3], Jun Wang [1,6], Jing Sun [1,6], Yi Li [1,*], Jyan-Chyun Jang [4,*], and Wenjing Li [2,5,*]

[1] Qinghai Provincial Key Laboratory of Qinghai-Tibet Plateau Biological Resources, Northwest Institute of Plateau Biology, Chinese Academy of Sciences, Xining 810008, China; yphu@nwipb.cas.cn (Y.H.); 848009178@qq.com (H.Z.); 54667402@qq.com (Q.Q.); SLnwipb@163.com (J.W.); qidian1518@sohu.com (J.S.); liyi@nwipb.cas.cn (Y.L.)
[2] Key Laboratory of Adaptation and Evolution of Plateau Biota, Northwest Institute of Plateau Biology, Chinese Academy of Sciences, Xining 810008, China; wjli@nwipb.cas.cn (W.L.)
[3] School of Life Sciences, Jinggangshan University, Ji'an 343009, China; 376664833@qq.com (G.L.)
[4] Department of Horticulture and Crop Science, The Ohio State University, Columbus 43210, OH, USA; Jang.40@osu.edu (J.J.)
[5] Scientific Research and Popularization Base of Qinghai-Tibet Plateau Biology, Qinghai Provincial Key Laboratory of Animal Ecological Genomics, Xining 810008, China
[6] University of Chinese Academy of Sciences, Beijing 100049, China
* Correspondence: wjli@nwipb.cas.cn (W.L.); liyi@nwipb.cas.cn (Y.L.); Jang.40@osu.edu (J.J.)

1. Supplementary Figures and Tables

1.1 Supplementary Figures

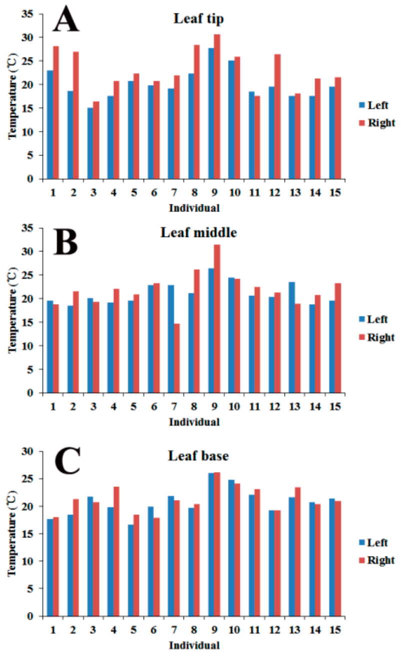

Figure S1. Leaf temperatures of 15 individual samples on the opposite sides of the middle of five first-order veins. (A) tip; (B) middle; (C) base.

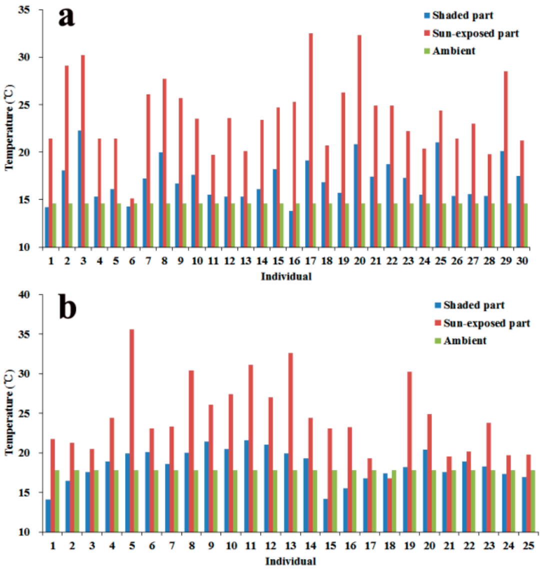

Figure S2. Leaf temperatures of sun-exposed and shaded parts from 25–30 individual samples. (a) ambient temperature at 14.6℃; (b) ambient temperature at 17.8℃.

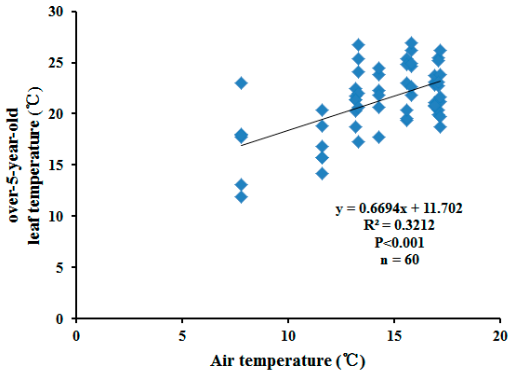

Figure S3. Correlation between leaf temperature of the over-5-year-old plant and air temperature during the time-course experiment.

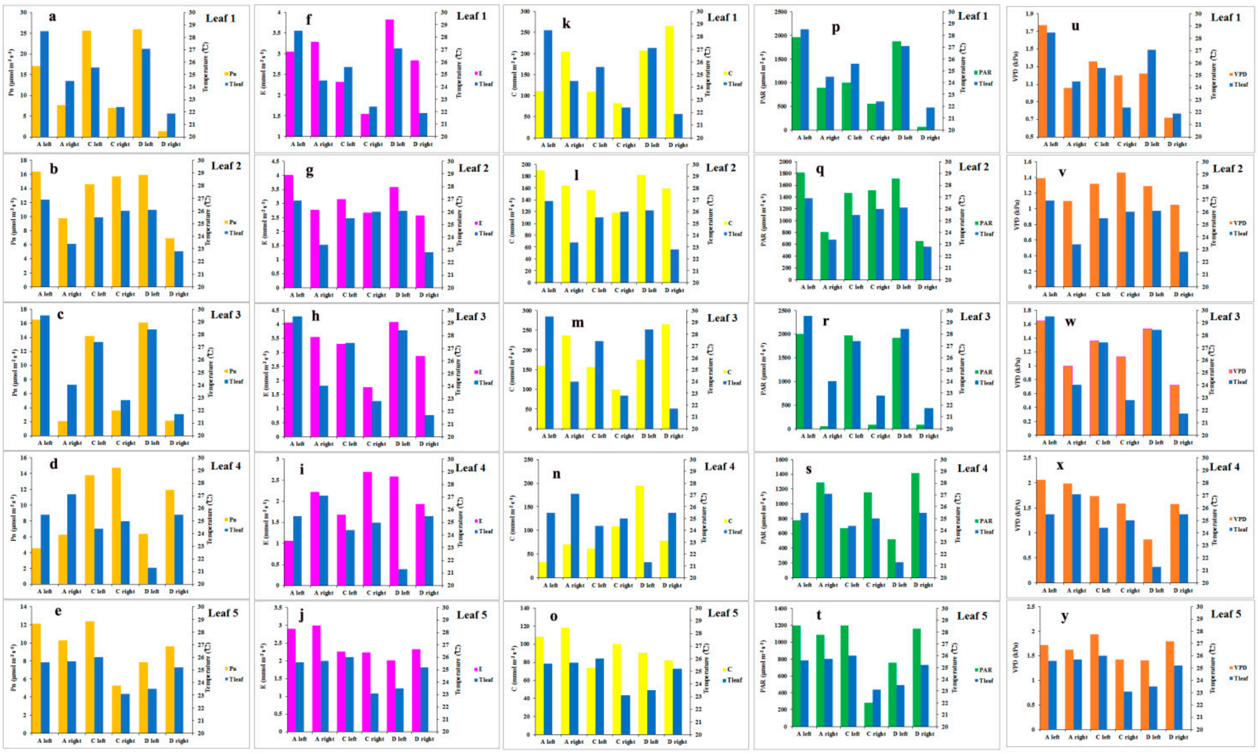

Figure S4. Leaf physiological parameters in different positions of large leaves from over-5-year-old plants in detail. (a-e) Net photosynthesis rate (Pn); (f-j) Transpiration rate (E); (k-o) Stomatal conductance rate (C); (p-t) Photosynthetically active radiation (PAR); (u-y) Vapor pressure deficit (VPD); Tleaf: leaf temperature; Leaf positions on two sides of the middle of five first-order veins are labeled (A left and A right) for the tip and (C left and C right) for the base; Leaf middle positions on the opposite sides of the fourth of five first-order veins are labeled (D left and D right).

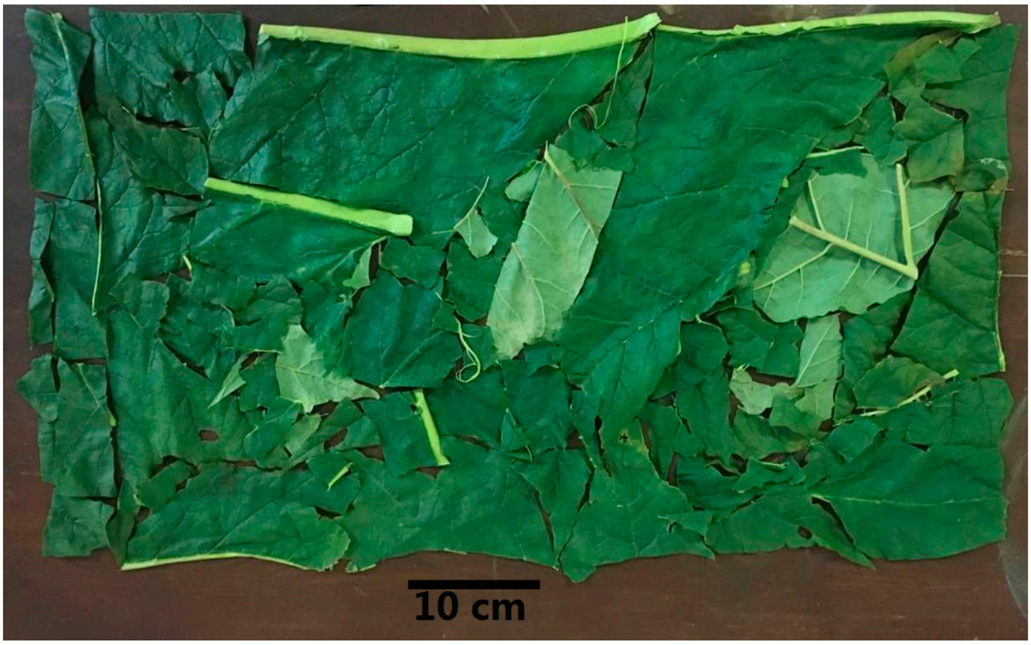

Figure S5. A spliced leaf composite from an over-5-year-old *R. tanguticum* plant.

1.2 Supplementary Tables

Table S1. Statistical analysis of the leaf characters of *R. tanguticum* at different ages.

	F	*d.f.*	*P*
Leaf Length	563.204	2,87	< 0.001
Leaf Width	580.901	2,87	< 0.001
Leaf area	88.471	2,59	< 0.001
Leaf dry mass	62.192	2,41	< 0.001
Leaf number per individual	217.963	2,91	< 0.001

One-way ANOVAs were performed with an alpha level of 0.01 or 0.05, and provided with the following *F* values (*F*) and degrees of freedom (*d.f.*).

Table S2. The angles between different first-order veins and plane of the middle of five first-order veins.

Traits	Value (°)
Between the plane of the middle of five first-order veins and second of five first-order veins	30.60 ± 2.44 (n = 5)
Between the plane of the middle of five first-order veins and first of five first-order veins	64.20 ± 4.43 (n = 5)
Between the second and third of five first-order veins	65.20 ± 2.27 (n = 5)
Between the first and second of five first-order veins	57.60 ± 2.50 (n = 5)
Between the first and fifth of five first-order veins	51.60 ± 8.87 (n = 5)

Table S3. Details of the calculations of intersection angle (α) and ratio of solar radiation exposure in leaves of over-5-year-old *R. tanguticum*.

Over-5-year-old	AB (mm)	AC (mm)	BC (mm)	AB^2	AC^2	BC^2	$AB^2+AC^2-BC^2$	2AB× AC	$\cos\alpha=(AB^2+AC^2-BC^2)/2AB\times AC$	α	BC/(AB+AC)
1	45	47	44	2025	2209	1936	2298	4230	0.5433	57.09	0.4783
2	36	35	35	1296	1225	1225	1296	2520	0.5143	59.05	0.4930
3	70	65	84	4900	4225	7056	2069	9100	0.2274	76.86	0.6222
4	52	50	41	2704	2500	1681	3523	5200	0.6775	47.35	0.4020
5	74	73	71	5476	5329	5041	5764	10804	0.5335	57.76	0.4830
6	70	70	66	4900	4900	4356	5444	9800	0.5555	56.25	0.4714
7	65	60	67	4225	3600	4489	3336	7800	0.4277	64.68	0.5360
8	61	59	55	3721	3481	3025	4177	7198	0.5803	54.53	0.4583
9	52	50	49	2704	2500	2401	2803	5200	0.5390	57.38	0.4804
10	52	52	52	2704	2704	2704	2704	5408	0.5000	60.00	0.5000
11	39	40	37	1521	1600	1369	1752	3120	0.5615	55.84	0.4684
12	40	42	37	1600	1764	1369	1995	3360	0.5938	53.58	0.4512
13	51	53	55	2601	2809	3025	2385	5406	0.4412	63.82	0.5288
14	40	39	41	1600	1521	1681	1440	3120	0.4615	62.51	0.5190
15	66	64	77	4356	4096	5929	2523	8448	0.2987	72.62	0.5923
16	39	40	44	1521	1600	1936	1185	3120	0.3798	67.68	0.5570
17	40	42	32	1600	1764	1024	2340	3360	0.6964	45.86	0.3902
18	57	60	55	3249	3600	3025	3824	6840	0.5591	56.01	0.4701
19	42	43	40	1764	1849	1600	2013	3612	0.5573	56.13	0.4706
20	83	80	85	6889	6400	7225	6064	13280	0.4566	62.83	0.5215
21	68	72	74	4624	5184	5476	4332	9792	0.4424	63.74	0.5286
22	33	35	37	1089	1225	1369	945	2310	0.4091	65.85	0.5441
23	40	42	40	1600	1764	1600	1764	3360	0.5250	58.33	0.4878
24	66	68	67	4356	4624	4489	4491	8976	0.5003	59.98	0.5000
25	70	68	78	4900	4624	6084	3440	9520	0.3613	68.82	0.5652
26	63	64	65	3969	4096	4225	3840	8064	0.4762	61.56	0.5118
27	47	46	43	2209	2116	1849	2476	4324	0.5726	55.07	0.4624
28	30	31	32	900	961	1024	837	1860	0.4500	63.26	0.5246
29	48	48	51	2304	2304	2601	2007	4608	0.4355	64.18	0.5313
30	47	42	49	2209	1764	2401	1572	3948	0.3982	66.54	0.5506
average										60.51	0.5033
SE										1.22	0.0091

Table S4. Details of the calculations of intersection angle (β) and ratio of solar radiation exposure in leaves of over-5-year-old *R. tanguticum*.

Over-5-year-old	DE (mm)	DF (mm)	EF (mm)	DE^2	DF^2	EF^2	$DE^2+DF^2-EF^2$	2DE×DF	$\cos\beta=(DE^2+DF^2-EF^2)/2DE\times DF$	β	EF/(DE+EF)
1	16	16	15	256	256	225	287	512	0.5605	55.91	0.4688
2	27	24	28	729	576	784	521	1296	0.4020	66.30	0.5490
3	28	23	22	784	529	484	829	1288	0.6436	49.94	0.4314
4	37	36	34	1369	1296	1156	1509	2664	0.5664	55.50	0.4658
5	27	27	27	729	729	729	729	1458	0.5000	60.00	0.5000
6	29	27	25	841	729	625	945	1566	0.6034	52.88	0.4464
7	26	28	27	676	784	729	731	1456	0.5021	59.86	0.5000
8	29	27	25	841	729	625	945	1566	0.6034	52.88	0.4464
9	20	21	20	400	441	400	441	840	0.5250	58.33	0.4878
10	27	29	28	729	841	784	786	1566	0.5019	59.87	0.5000
11	29	30	31	841	900	961	780	1740	0.4483	63.37	0.5254
12	30	30	29	900	900	841	959	1800	0.5328	57.81	0.4833
13	32	30	32	1024	900	1024	900	1920	0.4688	62.05	0.5161
14	16	15	17	256	225	289	192	480	0.4000	66.42	0.5484
15	42	41	39	1764	1681	1521	1924	3444	0.5587	56.04	0.4699
16	25	25	26	625	625	676	574	1250	0.4592	62.66	0.5200
17	31	32	30	961	1024	900	1085	1984	0.5469	56.85	0.4762
18	28	30	28	784	900	784	900	1680	0.5357	57.61	0.4828
19	32	30	31	1024	900	961	963	1920	0.5016	59.90	0.5000
20	29	30	33	841	900	1089	652	1740	0.3747	67.99	0.5593
21	34	36	40	1156	1296	1600	852	2448	0.3480	69.63	0.5714
22	27	26	30	729	676	900	505	1404	0.3597	68.92	0.5660
23	29	28	25	841	784	625	1000	1624	0.6158	51.99	0.4386
24	32	30	30	1024	900	900	1024	1920	0.5333	57.77	0.4839
25	41	43	45	1681	1849	2025	1505	3526	0.4268	64.73	0.5357
26	20	22	21	400	484	441	443	880	0.5034	59.77	0.5000
27	36	35	32	1296	1225	1024	1497	2520	0.5940	53.56	0.4507
28	29	28	28	841	784	784	841	1624	0.5179	58.81	0.4912
29	39	40	39	1521	1600	1521	1600	3120	0.5128	59.15	0.4937
30	33	32	34	1089	1024	1156	957	2112	0.4531	63.06	0.5231
Average										59.65	0.4977
SE										0.93	0.0070

Table S5. Details of the calculations of intersection angle (α) and ratio of solar radiation exposure in leaves of 3 to 4-year-old *R. tanguticum*.

3 to 4-year-old	AB (mm)	AC (mm)	BC (mm)	AB^2	AC^2	BC^2	$AB^2+AC^2-BC^2$	2AB ×AC	$\cos\alpha=(AB^2+AC^2-BC^2)/2AB\times AC$	α	BC/(AB+AC)
1	51	50	67	2601	2500	4489	612	5100	0.1200	83.11	0.6634
2	45	40	53	2025	1600	2809	816	3600	0.2267	76.90	0.6235
3	44	43	64	1936	1849	4096	-311	3784	-0.0822	94.71	0.7356
4	34	32	48	1156	1024	2304	-124	2176	-0.0570	93.27	0.7273
5	40	42	54	1600	1764	2916	448	3360	0.1333	82.34	0.6585
6	29	30	40	841	900	1600	141	1740	0.0810	85.35	0.6780
7	45	43	65	2025	1849	4225	-351	3870	-0.0907	95.20	0.7386
8	35	37	45	1225	1369	2025	569	2590	0.2197	77.31	0.6250
9	33	32	41	1089	1024	1681	432	2112	0.2045	78.20	0.6308
10	24	23	34	576	529	1156	-51	1104	-0.0462	92.65	0.7234
11	35	33	48	1225	1089	2304	10	2310	0.0043	89.75	0.7059
12	26	27	35	676	729	1225	180	1404	0.1282	82.63	0.6604
13	25	24	30	625	576	900	301	1200	0.2508	75.47	0.6122
14	31	30	43	961	900	1849	12	1860	0.0065	89.63	0.7049
15	24	23	28	576	529	784	321	1104	0.2908	73.10	0.5957
16	20	19	26	400	361	676	85	760	0.1118	83.58	0.6667
17	21	19	27	441	361	729	73	798	0.0915	84.75	0.6750
18	33	34	44	1089	1156	1936	309	2244	0.1377	82.09	0.6567
19	33	34	49	1089	1156	2401	-156	2244	-0.0695	93.99	0.7313
20	34	36	51	1156	1296	2601	-149	2448	-0.0609	93.49	0.7286
21	42	43	58	1764	1849	3364	249	3612	0.0689	86.05	0.6824
22	31	28	42	961	784	1764	-19	1736	-0.0109	90.63	0.7119
23	37	38	51	1369	1444	2601	212	2812	0.0754	85.68	0.6800
24	32	30	46	1024	900	2116	-192	1920	-0.1000	95.74	0.7419
25	18	18	25	324	324	625	23	648	0.0355	87.97	0.6944
26	31	33	44	961	1089	1936	114	2046	0.0557	86.81	0.6875
27	19	19	26	361	361	676	46	722	0.0637	86.35	0.6842
28	17	17	25	289	289	625	-47	578	-0.0813	94.66	0.7353
29	19	20	24	361	400	576	185	760	0.2434	75.91	0.6154
30	20	28	27	400	784	729	455	1120	0.4063	66.03	0.5625
Average										85.44	0.6779
SE										1.38	0.0087

Table S6. Summary of statistical analysis of the temperature of opposite parts of leaves.

	t	*d.f.*	*P*
Tip	4.51	1,14	< 0.001
Middle	0.86	1,14	0.41
Base	1.23	1,14	0.24
Sun-exposed vs shaded	14.33	1,54	< 0.001
Highest vs lowest	53.26	1,149	< 0.001

Paired-samples T-tests were used to analyze the temperature of opposite parts of leaf (right and left sides of the middle of five first-order veins, i.e., sun-exposed and shaded part), and highest vs lowest temperature of one thermal imaging of *R. tanguticum* leaf. The following *t* values (*t*) and degrees of freedom (*d.f.*) were provided. The *P* values were based on two-tailed permutation tests.

Table S7. Summary of statistical analysis of the photosynthetic parameters of opposite parts of over-5-year-old large leaves.

parameters	*t*	*df*	*P*
Pn	3.157	1,14	0.007
E	1.779	1,14	0.097
C	0.630	1,14	0.539
PAR	2.566	1,14	0.022
Tleaf	2.431	1,14	0.029
VPD	1.989	1,14	0.067

Paired-samples T-tests were used to analyze the photosynthesis of opposite parts of leaf (right and left sides of large leaves), The following *t* values (*t*) and degrees of freedom (*d.f.*) were provided. The *P* values were based on two-tailed permutation tests.

Table S8. Correlation matrix of photosynthetic parameters and environmental factors in *R. tanguticum*.

factor	Pn	E	C	Tleaf	PAR	VPD
Pn	1	0.405*	-0.100	0.683**	0.775**	0.305
E		1	0.699**	0.486**	0.526**	-0.250
C			1	-0.195	-0.119	-0.802**
Tleaf				1	0.900**	0.641**
PAR					1	0.491**
VPD						1

*. Correlation is significant at the 0.05 level (2-tailed).

**. Correlation is significant at the 0.01 level (2-tailed).

Table S9. Multiple linear regression equations for photosynthetic parameters and environmental factors of *R. tanguticum*.

Photosynthetic parameters	Multiple linear regression equations	Correlative coefficients *R*	*F*	*P*
Pn	Y = 2.821+0.775PAR	0.775	42.233	＜0.001
E	Y = -4.465+1.095Tleaf-0.951VPD	0.877	45.035	＜0.001
C	Y = 21.609+0.541Tleaf-1.149VPD	0.903	59.872	＜0.001

Table S10. Statistical analysis of the speed of airflow around the leaves of over-5-year-old plants.

	t	*d.f.*	*P*
Middle area vs outside area	5.29	1,59	< 0.001

Paired-samples T-tests were used to analyze the airflow in the middle area (approximately 1 cm above the middle of the third of five first-order veins) and outside area (10 cm vertical above the middle area) of the leaves of over-5-year-old plants. The following *t* values (*t*) and degrees of freedom (*d.f.*) were provided. The *P* values were based on two-tailed permutation tests.

2. Supplementary Video

Video S1. Airflow indicated by smoke movement around the large leaves of over-5-year-old plants.
